# Gut microbiota: The key to the treatment of metabolic syndrome in traditional Chinese medicine – a case study of diabetes and nonalcoholic fatty liver disease

**DOI:** 10.3389/fimmu.2022.1072376

**Published:** 2022-12-23

**Authors:** Yang Bao, Xiao Han, Da Liu, Zhaolin Tan, Yongzhi Deng

**Affiliations:** ^1^ Department of Endosecretory Metabolic Diseases, Affiliated Hospital of Changchun University of Chinese Medicine, Changchun, Jilin, China; ^2^ School of Pharmacy, Changchun University of Chinese Medicine, Changchun, Jilin, China; ^3^ Department of Acupuncture and Massage, The Third Affiliated Hospital of Changchun University of Chinese Medicine, Changchun, Jilin, China

**Keywords:** gastrointestinal flora, diabetes mellitus, non-alcoholic fatty liver disease, traditional Chinese medicine, inflammation, short chain fatty acids

## Abstract

Metabolic syndrome mainly includes obesity, type 2 diabetes (T2DM), alcoholic fatty liver (NAFLD) and cardiovascular diseases. According to the ancient experience philosophy of Yin-Yang, monarch-minister compatibility of traditional Chinese medicine, prescription is given to treat diseases, which has the advantages of small toxic and side effects and quick effect. However, due to the diversity of traditional Chinese medicine ingredients and doubts about the treatment theory of traditional Chinese medicine, the mechanism of traditional Chinese medicine is still in doubt. Gastrointestinal tract is an important part of human environment, and participates in the occurrence and development of diseases. In recent years, more and more TCM researches have made intestinal microbiome a new frontier for understanding and treating diseases. Clinically, nonalcoholic fatty liver disease (NAFLD) and diabetes mellitus (DM) often co-occur. Our aim is to explain the mechanism of interaction between gastrointestinal microbiome and traditional Chinese medicine (TCM) or traditional Chinese medicine formula to treat DM and NAFLD. Traditional Chinese medicine may treat these two diseases by influencing the composition of intestinal microorganisms, regulating the metabolism of intestinal microorganisms and transforming Chinese medicinal compounds.

## 1 Introduction

Intestinal microecology is an important part of human environment. It is composed of intestinal microbiota, intestinal epithelial cells and immune system, forms intestinal mucosal barrier and plays an important role in energy metabolism. Both external and genetic factors affect the composition and function of intestinal microecology. The steady state of microbiota is closely related to human health. There is growing evidence that intestinal microbiota and their metabolites play an important role in the development of obesity, diabetes and nonalcoholic fatty liver disease (1). Diabetes and non-alcoholic fatty liver are two diseases closely related to intestinal microbial homeostasis. T2DM is the main type of diabetes, mainly manifested as metabolic disorders, such as hyperglycemia, hyperlipidemia and insulin resistance. Diabetes can lead to a variety of serious complications, such as retinopathy and diabetic nephropathy, gestational diabetes, atherosclerosis and other cardiovascular diseases, these complications affect the quality of life of a large number of people around the world. The rapid growth of diabetes has brought a great burden on the global society and economic society (2). Nonalcoholic fatty liver disease (NAFLD), as a common chronic liver disease, can be divided into fatty liver, steatohepatitis and liver fibrosis according to the degree of inflammation and fibrosis. The main manifestations of NAFLD are steatosis, lipotoxicity and inflammatory injury, which are associated with glucose homeostasis and persistent low-grade inflammation (1) (3). Studies have found that intestinal flora and metabolites can reverse some metabolic disorders, including high fat, tissue inflammation and low insulin sensitivity and secretion (4). This suggests that intestinal flora can be used in the treatment of diabetes and fatty liver (5).

As an important supplementary means of clinical medicine, traditional Chinese medicine has been widely adopted in some East Asian countries. In some western countries, such as the United States, Britain and Germany, the trend of using traditional Chinese medicine as a treatment for diseases is becoming more and more obvious. Different from chemical drugs and biological agents, traditional Chinese medicine and traditional Chinese medicine formulations under the guidance of traditional Chinese medicine theory are often difficult to determine specific bioactive components. Traditional Chinese medicine prescribes prescriptions to treat diseases under the guidance of ancient empirical philosophy, such as yin-yang, monarch-minister compatibility and so on. Traditional Chinese medicine advocates the concept of wholeness and regards organs such as internal organs as a whole. the destruction of intestinal microbial homeostasis promotes the development of metabolic syndrome such as diabetes, fatty liver and cardiovascular syndrome, which is in line with the “whole” concept of TCM theory. There has been a sharp increase in patients with diabetes and non-alcoholic fatty liver disease worldwide, which studies have shown may be associated with insulin resistance. Patients usually suffer from these two diseases at the same time, which is a difficult problem in clinical treatment (6). As insulin resistance plays an important role in the development of non-alcoholic fatty liver, diabetes drugs are often used as the treatment option for the treatment of non-alcoholic fatty liver (7) (8). On the one hand: there are no approved drugs for nonalcoholic fatty liver disease, and the only approved treatment option is to improve diet and lose weight. On the other hand: the drugs used to treat diabetes are still defective in the treatment of fatty liver. With the discovery of plant-derived natural products quercetin, resveratrol, polysaccharides, berberine and curcumin in the treatment of diseases, researchers have focused on “simple, convenient and low-toxic” herbs. Researchers have found that single herbs such as Coptis chinensis, Radix Astragali, Ginseng and herbal formulations such as SiMiao, Gegen Qinlian decoction, Huanglian jiedu decoction and LLKL have potential therapeutic effects on T2DM and NAFLD. These herbs exert pharmacological effects through intestinal microflora and mainly include two ways: changing the composition of intestinal microorganisms and affecting the metabolism of intestinal microflora. The main purpose of this review is to explain the therapeutic effect of intestinal microbiota on diabetes and non-alcoholic fatty liver.

## 2 Association between diabetes mellitus and non-alcoholic fatty liver disease

### 2.1 Diabetes mellitus and non-alcoholic fatty liver disease – two clinically associated diseases

Diabetes is a metabolic disorder characterized by hyperglycemia caused by deficiency of insulin secretion and/or deficiency of insulin action. There are two main types of diabetes, insulin-dependent type 1 diabetes (T1DM) and insulin-independent type 2 diabetes mellitus (T2DM), of which type 2 diabetes accounts for 90% of patients with diabetes (9). Nonalcoholic fatty liver disease (NAFLD) is considered to be the most common form of liver disease in the world, including fatty liver, steatohepatitis (NASH) and liver fibrosis (10). NASH is a progressive form of nonalcoholic fatty liver. NAFLD is a risk factor for metabolic disorders such as obesity, diabetes, especially T2DM and cardiovascular disease. Among obese people undergoing bariatric surgery, the prevalence of NAFLD is as high as 90%, and among diabetics, the prevalence of NAFLD can be as high as 71%. Insulin resistance (IR) exists in almost all patients with NAFLD and T2DM. Through the evaluation of the homeostasis model of insulin resistance, it was found that there was a significant correlation between IR and the prevalence of steatohepatitis in NAFLD. The relationship between diabetes and nonalcoholic fatty liver gradually evolved into the relationship with simple steatosis (SS), NASH and liver fibrosis (11) (12). Cross-sectional studies have shown that non-alcoholic fatty liver disease usually occurs in patients with type 2 diabetes (13). A systematic review and meta-analysis of 27 clinical trials confirmed the direct relationship between fatty liver and the incidence of diabetes (14). The probability of developing diabetes is also different in different steatosis states. Follow-up results showed that the incidence of diabetes in patients without steatosis, intermittent steatosis and persistent steatosis increased by 5.1%, 14.1% and 27.1%, respectively. It can be speculated that early intervention of steatosis has a resistant effect on the development of diabetes (15) A study based on patients with nonalcoholic fatty liver disease and first-degree relatives in the United States found that familial aggregation of insulin resistance syndrome has a genetic susceptibility to supporting nonalcoholic fatty liver disease (16). Family history of diabetes, especially in non-diabetic patients, is associated with nonalcoholic steatohepatitis (NASH) and fibrosis in NAFLD (17). In addition, the occurrence and development of cardiovascular diseases such as obesity, retinopathy, renal failure, peripheral neuropathy and atherosclerosis are also related to diabetes (9).

### 2.2 Beneficial effects of various anti-diabetic drugs on non-alcoholic fatty liver disease

In the above part, we have explained the clinical correlation between NAFLD and T2DM. However, NAFLD does not have an explicitly approved drug, and the only approved treatment option is to change diet and exercise. IR plays an important role in the development of NAFLD, and many hypoglycemic drugs have been evaluated for the treatment of NAFLD. These drugs mainly include biguanides, glucagon-like peptide 1 receptor (GLP-1) agonists, peroxisome proliferator-activated receptor (PPAR) agonists and farnesoid X receptor (FXR) agonists. Metformin is known to improve lipid metabolism by activating adenylate-activated protein kinase (AMPK), an important regulator of energy metabolism (18). Metformin exerts the preventive effect of NAFLD by increasing AMPK phosphorylation, inhibiting macrophage polarization, reducing macrophage infiltration and the expression of pro-inflammatory cytokines (TNF- α, IL-1 β and IL-6), relieving liver inflammation and fat accumulation (19, 20). In addition, metformin alleviates fatty liver degeneration in obese mice by affecting the protein levels of CYP7B1 and CH25H, a cholesterol hydroxylase, to regulate cholesterol secretion and metabolism (21). Glucagon-like peptide-1 (GLP-1) is an enterotropic insulin secreted by intestinal endocrine L cells that regulates glucose regulation by slowing gastric emptying and glucose-dependent inhibition of glucagon secretion. GLP-1 can improve liver insulin sensitivity (22, 23)and enhance the direct effect of lipid hydrolysis and oxidation on liver (24–26). Lilarutide is a kind of GLP-1 analogue. Studies have shown that liralutide can reduce liver enzymes, including alanine aminotransferase (ALT) and aspartate aminotransferase (AST) (27). Lipopeptide is associated with liver lipid metabolism, total cholesterol (TC) and triglyceride (TG) (28). Pioglitazone belongs to PPAR- γ agonist and has insulin sensitizing effect (29). Pioglitazone reduces the accumulation of lipids in the liver (30) by improving fatty acid uptake and transport. Farnesoid X receptor (FXR) is a kind of nuclear receptor activated by bile acid, which is highly expressed in the liver and intestines and is related to bile acid and lipid metabolism (31). FXR agonists can reduce insulin resistance, improve lipid metabolism disorders, and alleviate fatty liver degeneration (32).

### 2.3 Summary

In conclusion, there is a close relationship between diabetes and nonalcoholic fatty liver disease. Metformin, liralutide and pioglitazone are used in the treatment of diabetes and drugs can be developed for the treatment of non-alcoholic fatty liver.

## 3 How does the gut microbiota influence T2DM and NAFLD

### 3.1 Intestinal microbes

Intestinal microecology is an extremely complex ecosystem, which is composed of intestinal microflora, intestinal epithelial cells and intestinal immune system. Intestinal microecology is regarded as an important “organ”, which plays an important role in regulating human metabolism (33). Intestinal microflora, also known as intestinal bacteria, is a complex microbial community living in the gastrointestinal tract of the human body in a symbiotic way, which mainly includes two phyla, thick-walled bacteria and Bacteroides (34). Diabetes and nonalcoholic fatty liver disease are metabolic diseases related to obesity (35). Obesity increases the risk of diabetes and NAFLD in humans (36, 37). Intestinal flora disorders have been repeatedly observed in these metabolic diseases, which seem to be related to changes in the proportion of thick-walled bacteria and actinomycetes in the intestines (38, 39). In patients with T2DM, it was observed that the abundance of Streptococcus faecalis and Rosobacter increased, while the abundance of Shigella and Bifidobacterium decreased (40). The decrease of microbial diversity and the increase of Prevotella abundance were observed in the feces of children with NAFLD. Today, unhealthy Western diets are promoting and aggravating the course of T2DM and NAFLD, which may be reduced or reversed by intestinal flora treatment (41–43).

### 3.2 Intestinal microbial metabolites

#### 3.2.1 SCFAs

Intestinal ecological disorders usually lead to changes in intestinal SCFAs levels. Short-chain fatty acids (such as acetate, propionate and butyrate) produced by intestinal microorganisms not only provide nutrition and energy for the host (44), but also participate in lipid metabolism and glucose metabolism through a variety of pathways (45), thus affecting the development of T2DM and NAFLD. It has been found that human cells respond to SCFAs mainly by activating G protein coupled receptor (GPR41,GPR43) and inhibiting histone deacetylase (HDAC) (46, 47). G protein coupled receptors are expressed in adipose tissue (48), liver (49) and pancreatic β cells (50). Acetate is an important substrate for fatty acid synthesis, and the increase of acetate will lead to the accumulation of triglycerides (51, 52). Propionate is an important precursor of gluconeogenesis, and an increase in propionate levels will promote gluconeogenesis in the liver (53). Acetate and propionate activate GPR43 receptors, inhibit insulin signal transduction in adipocytes, inhibit fat accumulation and promote lipid and glucose metabolism in other tissues (54, 55). Butyrate promotes the expression of gluconeogenesis-related genes in a cAMP-dependent manner. In addition, SCFAs stimulates intestinal endocrine cells to secrete glucagon-like peptide 1 (GLP-1) and YY peptide (PYY) through a GPR-dependent mechanism. These two hormones inhibit appetite, promote fat oxidation, promote insulin secretion and reduce glucagon, and inhibit hepatic steatosis and the development of diabetes (47, 56). In addition, propionate and butyrate can also act as HDAC inhibitors to induce increased PYY mRNA levels (57).

#### 3.2.2 TMAO, BAs and BCAAs

Trimethylamine N-oxide (TMAO) is a metabolite associated with diabetes, liver steatosis and other chronic diseases (58). TMAO is derived from intestinal microflora that metabolizes choline. Choline is converted to trimethylamine (TMA) through Flavin-containing monooxygenase, and TMA is converted into TMAO in the liver (59). It was found that TMAO accumulated in the serum of patients with T2DM and NAFLD (60–62). TMAO can play a role in NAFLD by changing bile acid metabolism (63). In addition, TMAO may induce pancreatic β-cell dysfunction and promote the pathogenesis of T2D (64). Bile acids include primary bile acids and secondary bile acids. Primary bile acids are synthesized by cholesterol in the liver. Primary bile acids enter the intestine and are converted into secondary bile acids by intestinal flora. As an important mediator of intestinal-liver crosstalk, bile acid mainly acts on two key receptors, farnesoid X receptor (FXR) and Takeda G protein-coupled receptor 5 (TGR5), and regulates glucose homeostasis and lipid metabolism (65, 66). Bile acid metabolism is associated with the onset and progression of type 2 diabetes and NAFLD (67). Bile acid chelating agents can inhibit FXR activity in intestinal L cells, promote the production and secretion of GLP-1, and improve blood glucose (68). It can also reverse hepatic steatosis, inflammation and fibrosis by interrupting intestinal bile acid reabsorption (69). Branched chain amino acids (BCAAs) are essential amino acids, including leucine, isoleucine and valine (70). Intestinal microflora can produce and degrade branched chain amino acids. The increase of host branched chain amino acids is related to metabolic fatty liver disease and diabetes (71, 72). Amino acid-induced insulin signal transduction damage and G protein coupled receptor involvement lead to insulin resistance and type 2 diabetes mellitus (73). Leucine affects glucose metabolism by activating rapamycin complex 1mTORC1 (74). Host circulating branched chain amino acids were positively correlated with higher cholesterol level, liver fat content and insulin resistance (IR) (75). However, some studies have found that supplementation of branched chain amino acids can reduce the expression of adipogenesis-related genes FAS and ACC in the liver and reduce fat accumulation in the liver of rats fed with high-fat diet (72, 76).

### 3.3 Intestinal permeability and inflammation

The intestinal barrier consists of mucin layer and epithelial cells. The destruction of intestinal barrier makes it easier for bacterial metabolites and inflammatory cytokines to enter the circulatory system, which is related to the occurrence of metabolic syndrome (77). It is known that secondary bile acid pass inhibits the expression of intestinal tight junction protein and increases intestinal permeability (78). The production of LPS results from the overgrowth of Gram-negative bacteria in the intestinal tract. LPS circulates through the portal vein to the liver to induce liver injury and inflammation (79, 80). The increase of intestinal permeability and inflammation induced by LPS is mediated by TLR4-dependent activation of ganglion (81). By inducing the activation of TLR4/NF- κ B signal pathway, LPS upregulates the levels of inflammatory factors such as TNF- α, IL-1 and IL-10, and promotes oxidative stress, resulting in insulin resistance and NAFLD (82, 83). Similarly, SCFAs reduces intestinal inflammation by inhibiting the LPS/NF-kappa B/TLR4 pathway (84). SCFAs reduces inflammation by inhibiting the activity of histone deacetylase (HDAC) and promoting the production of regulatory T cells (Treg) (85).

### 3.4 Summary

From the above introduction, it can be known that intestinal microbiota disorder is the key to the occurrence and development of T2DM and NAFLD. It can induce local organ or systemic inflammation by changing the diversity of intestinal flora, affecting microbial metabolism and destroying intestinal barrier.

## 4 Intestinal flora– the “target organ” of traditional Chinese medicine in the treatment of diseases

**Figure 1:**
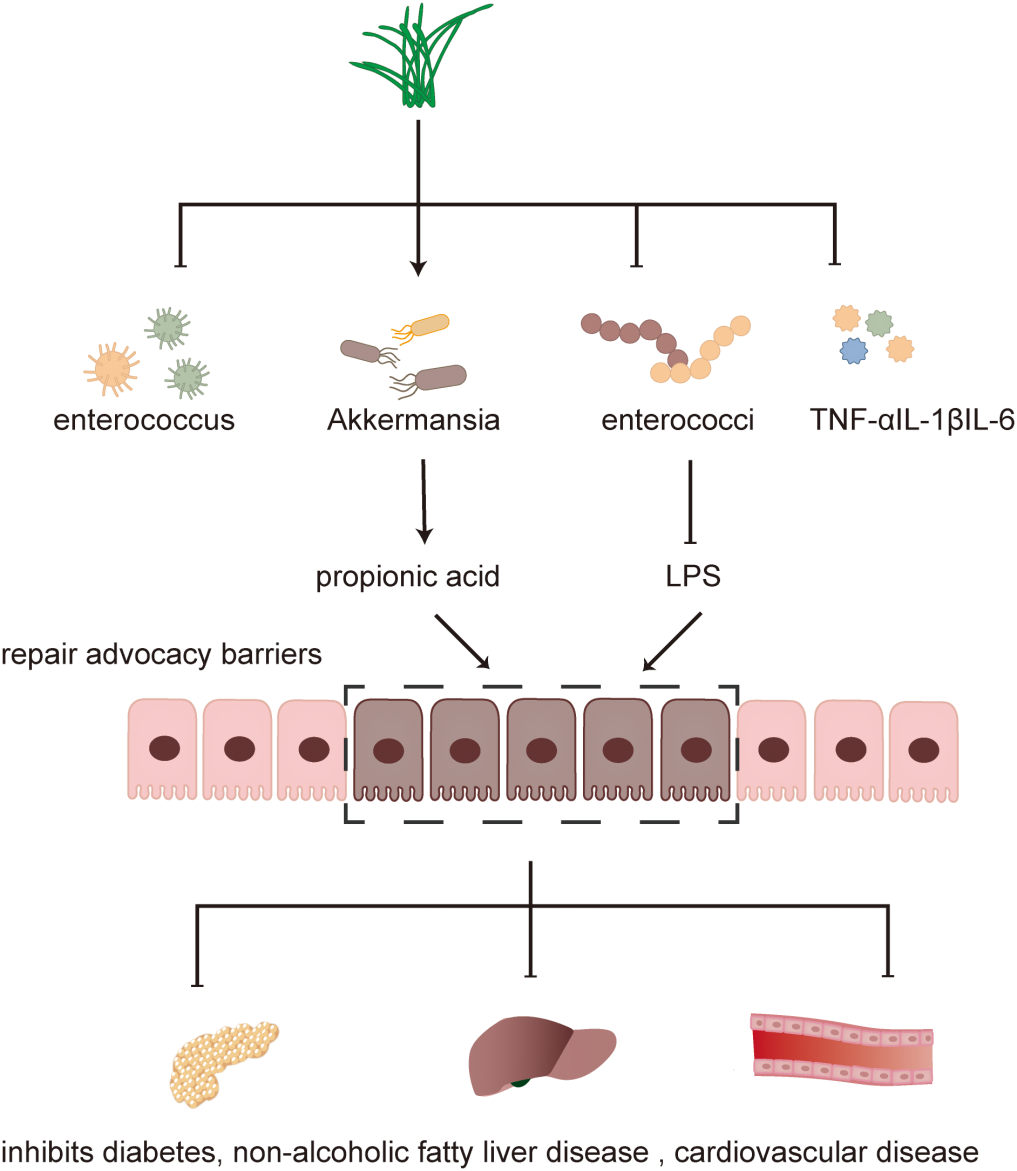
Mechanism of TCM in treating diseases.

**Figure 2:**
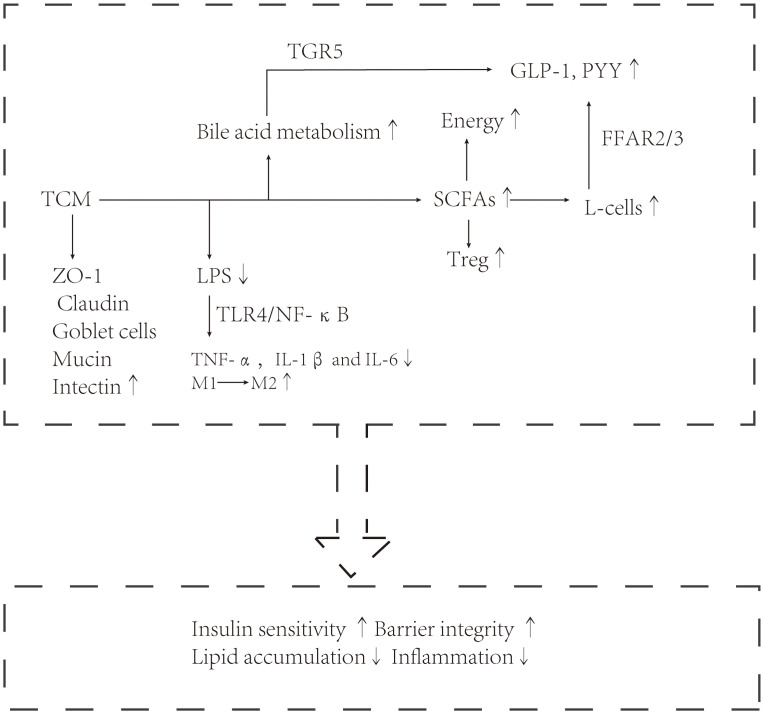
Traditional Chinese medicine exerts a therapeutic effect through intestinal flora, such as enterococcus, Akkermansia, and Vibrio desulfurization.

Traditional Chinese medicine has a history of treating diseases in China for more than 2000 years, including single drug treatment and compound drug treatment. T2DM and NAFLD are metabolic diseases characterized by hyperglycemia and fat accumulation. Intestinal flora mediates the occurrence and development of metabolic diseases and is used as an important organ to participate in metabolic regulation. A series of experimental results also show that the hypoglycemic and lipid-lowering effect of traditional Chinese medicine is related to intestinal flora. Below, we will introduce the molecular mechanism of traditional Chinese medicine in the treatment of diabetes and fatty liver from the point of view of intestinal flora structure, intestinal barrier and intestinal metabolites. The mechanism of the therapeutic effect of traditional Chinese medicine (TCM) is shown in **Figures 1**, **2**.

### 4.1 Individual herbs or herbal extracts

It is well known that traditional Chinese medicine extracts resveratrol, berberine, ginsenosides and curcumin play a beneficial regulatory role in lipid and glucose metabolism. Resveratrol is a natural polyphenol compound found in most herbs and has the potential to relieve diabetes and liver steatosis (86). It has been proved that the therapeutic effect of resveratrol is mediated by intestinal flora. For example, resveratrol alleviates the progression of diabetic nephropathy by reversing the low levels of Bacteroides, Alistipes, Rikenella, Odoribacter, Bacteroides and Alloprevotella in db/db mouse model. The therapeutic effect of resveratrol on db/db mice is related to resveratrol reversing the imbalance of intestinal flora, improving intestinal barrier, reducing intestinal permeability and inflammation (87). In addition, resveratrol can act as a potential NAFLD replacement therapy, and its therapeutic effect has been evaluated and confirmed in a number of trials. Previous experimental results have shown that resveratrol can improve lipid metabolism and reduce lipogenesis and inflammation in high-fat-fed mice, thus reducing hepatic steatosis (88). A new study found that high-fat diet (HFD)-induced NAFLD mice treated with resveratrol reduced the enrichment of lipid and glucose metabolism-related pathways, and this change was closely related to changes in intestinal flora. Resveratrol can reshape the diversity and composition of intestinal flora at different levels of the family. At the phylum level, the number of thick-walled bacteria increased significantly, while that of Bacteroides decreased significantly; at the family level, the erysipelaceae increased; at the genus level, the Olsenella content increased. Resveratrol reduces the invasion of harmful substances by up-regulating tight junction protein zo-1 and ameliorates liver inflammation by down-regulating inflammatory factors (IL-1, TNF- α, MyD88 and TLR-4) (89).

Berberine is a natural isoquinoline alkaloid extracted from herbal plants, which is the main activity of Coptis chinensis and Berberis (90, 91). The interaction between berberine and intestinal flora can alleviate metabolic disorders such as T2DM and NAFLD. Intestinal flora affects the absorption and transformation of berberine in gastrointestinal tract, and berberine also interferes with the structure and function of intestinal flora (92). In a study of Sprague-Dawley (SD) rats, the intestinal microflora diversity and richness of rats treated with berberine changed. At the gate level, there are higher abundance of Bacteroides and lower abundance of Proteus and verrucous microorganisms. At the family level, the family of Lactobacillus was significantly up-regulated. The concentrations of tyrosine, tryptophan and phenylalanine, the metabolites of intestinal flora, decreased in intestine and serum. Some studies have shown that high concentrations of aromatic amino acids are positively correlated with the risk of diabetes (93). Therefore, berberine treatment reduced the risk of diabetes in SD rats (94). Berberine is metabolized to berberine in the liver (95). It has been proved that berberine can regulate bile acid metabolism, activate intestinal farnesoid X receptor (FXR) and inhibit hepatic gluconeogenesis, and has significant lipid-lowering and hypoglycemic effects (95). Interestingly, by analyzing the composition of intestinal flora in high-fat (HFD)-fed mice, the researchers found that berberine increased intestinal beneficial bacteria, such as ileobacteria and myxobacteria. In addition, berberine reduced fat accumulation in the liver of HFD mice and decreased the levels of ALT and AST, which were beneficial to the treatment of NAFLD. Berberine can improve the imbalance of glucose homeostasis in HFD mice by affecting the expression of proteins related to glucose metabolism (PPAR γ, G6Pase, GLUT2,p-GSK) (96).

Ginsenosides are a kind of bioactive components extracted from plant medicine Ginseng. Ginsenosides can fight a variety of diseases through intestinal flora (97). Ginsenoside Rg1 can relieve T2D symptoms induced by HFD and streptozotocin (STZ) in rats, which may be related to the increase of the proportion of lactic acid bacteria and Lachnospiraceae and the decrease of the proportion of Lactobacillus by Rg1. Spearman correlation analysis showed that Lactobacillus was positively correlated with IL-1 β, IL-6, TNF- α and ROS levels (98). Lachnospiraceae is the main source of intestinal SCFAs, especially butyric acid (99, 100). Rg5 relieves inflammation by reducing plasma LPS levels and inhibiting the activation of TLR4-related signaling pathways in db/db mice. The hypoglycemic effect of Rg5 is related to its reducing the abundance of thick-walled bacteria and verrucous microorganisms and increasing the abundance of Bacteroides and Proteus in db/db mice (101). 25-hydroxy-protopanaxatriol (T19) is a new type of ginsenoside. Lachnospiraceae is a beneficial bacteria that regulates glucose and lipid metabolism. It was found that T19 significantly improved the abnormal glycolipid levels induced by HFD and STZ by significantly increasing the relative abundance of Lachnospiraceae family (102).

Curcumin is a polyphenol compound, mainly found in turmeric root (103). Curcumin attenuates dextran sulfate-induced T2MD symptoms in mice by reshaping the balance of Th17 and Treg in lymphoid cells. Th17 and Treg are related to the secretion of pro-inflammatory factor IL-17A and anti-inflammatory factor IL-10, respectively. Spearman analysis showed that curcumin mainly relieved chronic inflammation caused by T2MD by increasing the level of Roseburia and decreasing the levels of Erysipelatoclostridum and norank_f_Oscillospiraceae (104). Tetrahydrocurcumin (THC) is the main metabolite of curcumin. THC improves diabetes in db/db mice by reducing the relative abundance of Proteus and actinomycetes and promoting the expression of GLP-1 in the pancreas (105).

Herbs such as Polygala, licorice, Scutellaria baicalensis and Lycium barbarum also have the potential to treat diabetes and chronic liver disease. Polygala polygala extract (PTE) inhibits fat accumulation by promoting the expression of PPAR α. In addition, PTE regulates metabolism by enriching Proteus and reducing deferrifying bacteria (106). Licorice extract can reduce intestinal inflammation by reducing the levels of NF- κ B, Toll-like receptor 4 (TLR4) and tumor necrosis factor-α (TNF- α) in the colon of diabetic mice. The recovery of intestinal microbiology by licorice extract is related to the decrease of Lachnospiraceae _ NK4A136 content at genus level (107). The water extract of Scutellaria baicalensis Georgi can treat diabetes and complications by regulating the interaction between intestinal flora and bile acid metabolism. FXR is highly expressed in liver and intestine and is the key receptor of bile acid. Scutellaria baicalensis water extract can inhibit the expression of FXR in diabetic rats. Water extract of Scutellaria baicalensis Georgi can reverse the low levels of thephylaTenericutes and Patescibacteria and decrease the abundance of Lactobacillus and feacalibaculum in diabetic rats (108). Lycium barbarum polysaccharides can increase the proportion of probiotics, such as Ackermania, Lactobacillus and Prevaceae; Lycium barbarum polysaccharides can reduce intestinal pH and regulate the intestinal environment; Lycium barbarum polysaccharides can also stimulate innate immunity in the intestinal mucosa, such as macrophages or lymphocytes (109, 110). In addition, some Chinese herbal and natural plant extracts, such as cinnamon, Dendrobium, Radix Astragali, rhubarb, Aristolochia manshuriensis, cichoric acid, inulin, polyphenols, Ganoderma lucidum and mulberry polysaccharides are also effective in preventing and treating T2DM, NAFLD and related metabolic diseases. More details are shown in **Table 1**.

**Table 1:** The mechanism of action of individual herb or herbal extracts.

Herb/Extract	Subjects	Results	Gut microbiota	Mechanisms	References
Resveratrol	db/db mice	BW, FBS↓	Bacteroides, Alistipes, Rikenella, Odoribacter, Parabacteroides, and Alloprevotella genera↑	1.Gut barrier: ZO-1 2.Inflammation: LPS, IFN-g, TNF-a, IL-6↓ 3.Gut–kidney axis	(87)
Resveratrol	SD rats	TG, T-CHO↓	Akkermansia muciniphila, Ruminococcaceae, and Lachnospiraceae↑; Desulfovibrio↓	1.Gut barrier: occludin, ZO1, claudin1↑; the endocannabinoid system(CB1) ↓ 2.Inflammation: FAK, MyD88, and IRAK4↓; the endocannabinoid system(CB2) ↓	(111)
Resveratrol	C57BL/6J mice	BW,AST, TG,CHOL, LDL-C↓; GSH↓	Olsenella,Hydrogenoanaerobacterium↑; Barnesiella, Parasutterella↓	1.Gut barrier: zo-1, occludin↑ 2.Oxidative stress↓ 3.Inflammation: TLR4, MyD88, IL-1, TNF-α↑ 4. Fatty acid metabolism: Fabp2, Fabp1, Cpt1, Acox1↓	(89)
Berberine	SD rats	HOMA-IR,OGTT, FBG↓	Lactobacillaceae↑; Proteobacteria, Verrucomicrobia↓	1.Energy metabolism: amino acids (AAAS) and lipids	(94)
Berberis kansuensis	Wistar rats	BW、FBG、GSP、HOMA-IR↓	phyla Bacteroidetes, genera Akkermansia↑	1. inflammation: LPS, TNF-α, IL-1β,IL-6 2. IR and IS	(112)
Berberrubine	C57BL/6J mice	BW,ALT, AST↓	Ileibacterium,Mucispirillum↑	1. lipid metabolism: ACC1,FAS,CD36↓; ATGL, GK,PPAR-α,CPT-1↑	(96)
Rg1	SD rats	BW, FBG, TC, TG, LDL-C, HOMA-IR↓; LDL-C, HOMA-IS↑	Lachnospiraceae_NK4A136_group, Lachnoclostridium↑	1. SCFAs 2. Oxidative stress: 3. IR and IS	(98)
Rg5	db/db mice	FBG, OGTT↓	Firmicutes, Verrucomicrobia↓	1.Gut barrier: Occludin, ZO-1 2.Inflammation: LPS/TLR4	(101)
T19	HepG2, HFD/STZ mice	FBG, TG, TC, LD↓; BW, HDL↑	Lachnospiraceae↑	1. Insulin Signal Pathway: AMPK and PI3K	(102)
Curcumin	C57BLKS/J(−/−)_mice	Blood glucose↓	Roseburia, Erysipelatoclostridum, norank_f_Oscillospiraceae	1. Th17/Treg: IL-17A, IL-10	(65)
Curcumin	specificpathogen-free(SPE) rats	BW, HOMA-IR↓	Bacteroidetes, Bifidobacterium↑ Enterobacterales, Firmicutes↓	1. Gut barrier: occluding, ZO-1 2.Insulin resistance 3. Inflammation: LPS, TNF-α, TLR4/NF-κB	(113)
Tetrahydrocurcumin	C57BL/6 J mice	Serum insulin and pancreatic GLP-1↑	Firmicutes↑, Actinobacteria↓	1. GLP-1	(105)
Polygala tenuifolia	ICR mice	BW, ALT, AST, triglycerides, glucose↓	Proteobacteria↑, Deferribacteres↓	1. Lipid and cholesterol biosynthesis: PPARα	(106)
Radix Scutellariae	SD rats	FBG, LDL-C, OGTT, HOMA-IR↓	phyla Tenericutes, Patescibacteria↑, Lactobacillus, feacalibaculum↓	1. Bile acid metabolism: CYP7A1	(108)
Lycium barbarum polysaccharides	C57BL/6 mice	BW, TC, TG, LDL-C↓	Proteobacteria↓, Lactobacillus spp↑	1. SCFAs	(110)
Lycium barbarum L. leaves	SPF-grade rats	FBG, TCHO, TG, LDL-C, FFA, ALT, AST, a↓	Marvinbryantia, Parasutterella, Pre- votellaceae_NK3B31_group, Blautia, Ruminococcus_1, Coprococcus_2	1. Nicotinate and nicotinamide metabolism 2. Arachidonic acid metabolism	(114)
Cinnamaldehyde	C57 mice	OGTT, IPITTs, IGF1R, IRS1↓	Lactobacillus johnsonii↑, Lactobacillus murinus↓	1. Bile acid metabolism:Deoxycholic acid/FXR/AMPK 2. Insulin sensitivity	(115)
Dendrobium	db/dbmice	BW, LDL-C, MDA↓ INS, SOD, CAT, GSH↑	Bacteroidetes/Firmicutes, Prevotella /Akkermansia, S24-7/Rikenella/Escherichia coli	1. Lipid metabolism 2. Inflammation 3. Oxidative stress	(116)
Astragaloside IV	Kunming mice	TG, LDL, MDA↓, HDL, SOD↑	Pelatoclostridum↑, Bacteroides, Oscillibacter, Parabacteroides, Roseburia↓	1. Signaling pathways: AMPK/SIRT1, PI3K/AKT 2.SCFAs: Butyric acid 3. Oxidative stress 4.Lipid metabolism	(117)
Astragaloside IV	C57BL/6 mice	TC, TG, LDL-C,ALT, AST↓ GLP-1, HDL-C↑	Bacteroides, Lactobacillus, Streptococcus, Enterococcus, Lactococcus↓	1. Bile acid metabolis: FXR	(118)
Laminaria japonica polysaccharide	C57BL/6 mice	ITT, OGTT, HOMA-IR↓	Akkermansia	1. Insulin resistance 2.Inflammation:LPS, TLR4	(119)
Mulberry fruit polysaccharide	db/dbmice	TG, LDL-C, MDA, FFA ↓HDL-C, SOD, GSH-Px, CAT↑	Bacteroidales, Lactobacillus, Allobaculum, Bacteroides, and Akkermansia↑	1. Lipid metabolism	(120)
Chicoric Acid	C57BL/6 mice	BW, TC, TG, LDL-C, ROS, GPT-ALT, GOT-AST↓MDA, HDL-C IL-10	Lactoba- Callus, Turicibacter, Ruminococcaceae_ UCG-014, Alloprevotella, Candidatus_Saccharimonas	1. Signaling pathway: AMPK/Nrf2/NFκB	(121)
Inulin	C57BL/6 mice	ALT, AST, OGTT, HOMA-IR↓	Akkermansia, Bifidobacterium↑ Firmicutes/Bacteroidetes↓	1.SCFAs 2.Inflammation: (IL)-18, IL-1β, TNF-α, IL-6↓, IL-10↑	(122)
Rhubarb	C57BL/6J mice	BW, FBG, OGTT, IR, TC, TG, LDL-C↓	*Akkermansia muciniphila*	1. Insulin resistance 2. Inflammation: RANTES, TNF-α, IL-6, IFN-γ 3. Lipid metabolism	(123)
Akebia saponin D	C57BL/6J mice	FBG, TC, TG, LDL-C, HOMA-IR↓	Alistipes, Prevotella↓ Butyricimonas, Ruminococcus, Bifidobacter↑	1.Signaling pathway: PPAR-γ/FABP4	(124)
Green Tea Polyphenols	C57BL/6J mice	TC, TG, LDL-C, INS↓	Bacteroidetes/Firmicutes	1.SCFAs: Acetic acid, butyric acid↑ 2.Lipid metabolism	(125)
Quercetin	C57BL/6J mice	BW, FBG, HOMA-IR↓	Akkermansia, Verrucomicrobia phylum↑	1. Lipid metabolism 2. Inflammation: TLR-4, NLRP3, TNF-α 3. SCFAs: Butyrate	(126)
Ganoderic acid A	Kunming mice	TC, TG, LDL-C, AST, ALT, MDA↓ SOD, GSH↑	Lactobacillus, Burkholderia_Caballeroria_Paraburkholderia, Escherichia_ Shigella, Erysipelatoclostridium↓Aerococcus, Bilophila, Bifidobacterium↑	1.Lipid metabolism 2. Inflammation	(127)
Ganoderma lucidum polysaccharides	SD rats	TC, TG, LDL-C, MDA ↓HDL-C, SOD, GSH↑	Proteus, Ruminococcus, Coprococcus↓	1.SCFAs: Acetic acid, propionic acid, butyric acid 2. Inflammation: IL-1β, IL-6	(128)
Morchella esculenta mushroom polysaccharide	BALB/c mice	BW, FBG, INS, HOMA-IR↓	Lactobacillus↑ Corynebacterium, Facklamia↓	1.Bile acid metabolis 2. Inflammation: IL-6, IL-1β, TNF-α	(129)
laurolitsine	db/db mice	FBG, TC, TG, LDL-C↓ HDL-C↑	Mucispirillum schaedleri, Anaerotruncus_sp_G3_2012↓	1.Signaling pathway: LKB1-AMPK 2. Inflammation: IL-1β, TNFα, IL-6, IL-18, IL-10 3.Lipid metabolism	(130)
Gynostemma pentaphyllum	SD rats	FBG, TC, TG, LDL-C, ALT, AST, HOMA-IR↓HDL-C↑	Elusimicrobia, Cyanobacteria, Lactococcus spp↑ Ruminococcus spp↓	1. Lipid metabolism 2. Gut barrier 3.Inflammation : TNF-α, IL-1β, IL-6, TLR4	(131)
Gynostemma pentaphyllum polysaccharides	C57BL/6 mice	TC, TG, LDL-C, ALT, AST↓ HDL-C↑	Lactobacillus, Akkermansia↑Clostridia_ uncultured↓	1.Signaling pathway: TLR2/NLRP3	(132)
Poria cocos polysaccharides	C57BL/6 mice	TC, TG, LDL-C, ALT, AST, MDA↓ HDL-C↑	Faecalibaculum, Escherichia_Shigella, unclassified Oscillospirales↑ Tuzzerella, Enterococcus, Staphylococcus↓	1. Signaling pathway: NF-κB/CCL3/CCR1	(133)
Astragalus mongholicus polysaccharides	SD rats	WB, TC, TG, LDL-C,ALT, AST, HOMA-IR↓ HDL-C↑	Proteobacteria, Epsilonbacteria↑Firmicutes/Bacteroidetes↓	1. Signaling pathway: AMPK-PPAR-α, TLR4 - NLRP3, SCFAs-GPR 2. Gut barrier: ZO-1, Occludin	(134)
Pueraria lobata starch	C57BL/6J mice	TC, TG, LDL-C, ALT, AST↓	Lactobacillus, Bifidobacterium, Turicibacter↑Desulfovibrio↓	1. SCFAs 2. Lipid metabolism 3. Inflammation: IL-6, TNF-α	(135)
Salviae polysaccharide	C57/BL6 mice	BW, FBG, TC, TG, LDL-C↓	Ruminococcus_gnavus, Clostridium_cocleatum, Bifidobacterium_pseudolongum↓	1. Lipid metabolism 2. Inflammation: IL2, IL10, TGF-β, IL-6, IL23 3. Gut barrier:LPS	(136)
Nuciferine	SD rats	Conjugated BA, Non-12OH BA↑ TC, TG↓	Akkermansiaceae, Akkermansia, norank_f_Erysipelotrichaceae, Lachnospiraceae_NK4A136_group↑	1. Bile acid metabolis	(137)
Nuciferine	SD rats	BW, TC, TG, LDL-C↓ HDL-C↑	Akkmensia muciniphila, Ruminococcaceae, Desulfovibrionaceae	1. Signaling pathway:TLR4/MyD88/NF-κB 2. Gut barrier: ZO-1, Occludin, Mucin2 3. SCFAs: Acetic acid, Propionic acid	(138)
Myristica fragrans	C57BL/6J mice	TC, TG, LDL-C↓	Akkermansia, Blautia, Bifidobacterium, Adlercreutzia↑	1. Signaling pathway:AhR-FAS, NF-κB	(139)

### 4.2 Chinese herbal formulae

The formula of traditional Chinese medicine is another important means for the treatment of diseases in traditional Chinese medicine, and it is often used in the diagnosis and treatment of clinical diseases as a supplement to western medicine. The compatibility of traditional Chinese medicine is not random. On the contrary, it is necessary to follow the principle of compatibility of traditional Chinese medicine and the principle of diagnosis and treatment of traditional Chinese medicine (140).

Pi-Dan-Jian-Qingdecoction (PDJQ) contains Radix Astragali, Radix Pseudostellariae, Coptis chinensis, Scutellaria baicalensis, Rhizoma Atractylodes, Salvia miltiorrhiza and Litchi. PDJQ has a good intervention effect on the clinical treatment of diabetes. In addition to regulating intestinal flora and inhibiting inflammation, the mechanism of PDJQ in treating diabetes is also related to the regulation of tryptophan metabolism, histamine metabolism and tricarboxylic acid (TCA) circulation. The specific results were as follows: at the genus level, PDJQ increased the relative abundance of Lactobacillus, Brucella, Bacteroides, Vibrio Desulfuricus and Ackermania, and decreased the relative abundance of Prevos. In addition, correlation analysis showed that the regulatory effects of PDJQ on tryptophan metabolism, histidine metabolism and TCA cycle pathway were related to the abundance changes of Lactobacillus, Bacteroides and Ackermann bacteria (141).

Gegen Qinlian Decoction (GQD) is composed of seven traditional Chinese medicines: Pueraria lobata, Coptis chinensis, Scutellaria baicalensis, Anemarrhena anemarrhena, American ginseng, red peony root and dried ginger. The mechanism of GQD in the treatment of diabetes is similar to that of berberine. GQD restores glucose homeostasis by increasing butyrate-producing bacteria, such as Faecalibacterium and Roseburia (142).

LingguiZhugan (LGZG) formula, a traditional Chinese medicine formula composed of Poria cocos, cassia twig, Atractylodes macrocephala and licorice, plays a useful role in the treatment of obesity-related diabetes. LGZG plays a role in controlling blood glucose and reducing insulin resistance, which may be mediated by intestinal microorganism OscillospiraandHelicobacte (143).

The effective components of inQiJiangtangTablet (JQJT) tablets are berberine, chlorogenic acid, astragalus polysaccharides and astragaloside IV mainly from Coptis chinensis, astragalus membranaceus and honeysuckle. Studies have shown that these active components are related to intestinal bacteria relieving insulin resistance and low-grade host inflammation. JQJT can increase the concentration of SCFAs in T2DM mice, especially butyric acid. JQJT treatment group showed lower desulphurization vibrio and higher Ackermania (144).

Xiexin T ang was first recorded in the synopsis of the Golden Chamber, an ancient Chinese medical book, and consists of rhubarb, Scutellaria baicalensis and Coptis chinensis. In traditional medicine, diabetes is called diabetes. Xiexin T ang has a long history in the treatment of diabetes and its effect is obvious. The new study found that Xiexin T ang improved diabetic symptoms in rats by changing the levels of bacteria that produce SCFAs and anti-inflammatory bacteria, such as Adlercreutzia, Barnesiella, and Prevotellaceae NK3B31 group (145).

In addition, other TCM formulations and TCM preparations derived from TCM formulations, such as Simiao Wan, Qijian mixturen, Naoxintong capsule and Herbal formula LLKL, have also been found to play a therapeutic role through intestinal flora. More details are shown in **Table 2**.

**Table 2:** The mechanism of action of Chinese Herbal Formulae.

Herbal Formula	Subjects	Results	Gut microbiota	Mechanisms	References
Pi-Dan-Jian-Qing decoction	SD rats	TG, TC, LDL, ALT, AST, MDA, HOMA-IR↓ HDL, SOD, GSH-Px↑	Prevotella↓ Lactobacill, Desulfovib, Akkerman, Bacteroides↑	1.Histamine metabolism 2.Tryptophan metabolism 3. TCA cycle 4.Oxidative stress 5. Inflammation	(141)
Gegen Qinlian Decoction	GK rats	BW, NFBG, HOMA-IR↓	Faecalibacterium, Roseburia↑	1.SCFAs: butyrate 2. Inflammation: IL-1β, IL-6, IL-17, TNF-α, IFN-γ, MCP-1 3. Lipid metabolism	(142)
Linggui Zhugan	C57BL/6 J mice	BW, FBG, TG, TC, LDL, FFA, HOMA-IR↓ HDL↑	Lactobacillus, Bacteroides↑ Helicobacter↓	1.Lipid metabolis 2.Insulin resistance	(143)
JinQi Jiangtang Tablet	C57BL/6J mice	FBG, HbA1c↓	Akkermansia↑ Desulfovibrio↓	1.SCFAs:Acetic acid, Propionic acid, Butyric acid 2. Insulin resistance: TNF-α, IL-6, MCP-1	(144)
Xiexin Tang	SD rats	TC, TG, LDL-C↓ HDL-C↑	Adlercreutzia, Alloprevotella, Barnesiella, Prevotellaceae NK3B31 group	1.Lipid metabolis 2. Inflammation:	(145)
Xiexin Tang	SD rats	TC, TG, LDL-C↓ HDL-C↑	Adlercreutzia Barnesiella, Blautia, Lachnospiraceae, Prevotellaceae NK3B31 group↑	1. SCFAs 2.Energy metabolism 3. Signal Pathway:PGC-1α/UCP-2, AMPK/mTOR	(146)
Simiao Wan	C57BL/6J mice	Primary BAs↑ Secondary BAs↓	Allobaculum, Clostridium, Akkermansia, Lactobacilus, Bilophila↑ Coprococcus, Halomonas↓	1. Bile acid metabolism	(147)
Si Miao	C57BL/6 mice	BW, ALT, AST, TC, LDL-C↓ HDL-C↑	Akkermansia, Bifidobacterium, Faecalibaculum↑	1.Lipid metabolism 2.Inflammation 3.Gut barrier	(10)
Qijian mixture	KKay mice	FBG, WB, TC, INS↓	Bacteroidetes, Lachnospiraceae NK4A136 group, Enterorhabdu, Lachnospiraceae, Prevotellacea, Parabacteroides↑	1.Signal Pathway: TP53, AKT1 and PPARA	(148)
Naoxintong capsule	SD rats	TG, TC, FFA, LDL-C↓ HDL-C↑	[Ruminococcus] gnavus group, Erysipelatoclostridium, Oscillibacter, Ruminiclostridium 9, Ruminococcus 1	1. Insulin resistance 2. Inflammation: IL-1β, TNF-α, IL-6↓ IL-4↑ 3. Lipid metabolism	(149)
LLKL	Zucker rats	FFA, TC, TG↓	Proteobacteria, Actinobacteria.	1. Signal Pathway: TLR4, MyD88, CTSK 2. Lipid metabolism 3. Inflammation: LPS, TNF-α, IL-6↓	(150)
Huang-Lian-Jie-Du-Decoction	SD rats	ALT, AST, TG, TC, LDL-C, HOMA-IR↓ SOD, CAT, GSH↑	Parabacteroides, Blautia, Akkermansia	1. SCFAs 2. Bile acid metabolism 3. Lipid metabolismI	(151)

## 5 Discussion

Traditional Chinese medicine can affect the abundance of intestinal microbiota at different levels (**Tables 1**, **2**). Therefore, we believe that the role of traditional Chinese medicine in the treatment of T2DM and NAFLD is probably related to its role in mediating intestinal microbial changes. There are differences in intestinal flora changes and therapeutic mechanisms mediated by different Chinese medicines.

### 5.1 Intestinal barrier

The damage of intestinal mucosa and the increase of inflammatory factors are related to T2DM and T2DM related metabolic diseases. Traditional Chinese medicine alleviates metabolic inflammation by increasing intestinal mucus and tight connection (152).Restoratol, ginsenoside Rg5, Curcumin, Nuciferinehe and traditional Chinese medicine formula Si Miao maintain the integrity of intestinal barrier by promoting the expression of tight junction protein ZO-1 and blocking protein. Nuciferine also enhances the intestinal barrier by increasing the expression of goblet cells and mucin2 (138). intestinal epithelium from damage by producing certain enzymes in the intestine. Resveratrol, Inulin, Rhubarb, Quercetin, and traditional Chinese medicine formulas such as Simiao Wan, JinQi Jiangtang T ablet, Huang Lian Jie Du Division can increase the abundance of Akkermansia. Escherichia coli is not conducive to maintaining the integrity of the intestinal barrier. The metabolic enzyme StcE produced will break down mucin, increase intestinal permeability and induce intestinal inflammation. Dendrobium can reduce the content of Escherichia coli in the intestine of db/db mice (116). Oscillibacter belonging to Ruminal Cocci family can also increase intestinal permeability. Astragaloside IV inhibits the increase of intestinal permeability by reducing the abundance of Oscillibacter (117).

### 5.2 Inflammation

LPS entering the intestinal tract will induce intestinal inflammation, and LPS mainly comes from vibrio desulfuricus (153). Inverterol, Pueraria lobata starch and Nuciferine can reduce the abundance of harmful bacteria, Vibrio desulfurization. LPS combines with TLR of intestinal epithelial cells to induce the release of proinflammatory factors and aggravate the host’s inflammatory response. Berberis kansuensis, Rhubarb, Quercetin, Morchella esculenta mushroom polysaccharide, Gynostemma pentaphyllum, Pueraria lobata start, as well as Chinese herbal formula Gegen Qinlian Reaction and JinQi Jiangtang T ablet can reduce TNF- α, IL-1 β And IL-6 levels, thereby relieving inflammation caused by bacterial endotoxin. Salviae polysaccharide, Laurolitsine, Inulin and Curcumin can increase the level of anti-inflammatory factor IL-10 (104, 122, 130, 136). In addition, Restoratol, Curcumin, Nuciferine and Chinese herbal formula LLKL can reduce TLR4/MyD88/NF- κ B pathway inhibits LPS induced inflammatory mediator production (89) (113, 138). In particular, Curcumin alleviates T2DM symptoms by maintaining the balance of immune cells Th17 and Treg, reducing intestinal mucosal damage and infiltration of inflammatory cells (104). Chiric Acid and laurolitsine regulate AMPK/NF- κ B signal pathway can reduce systemic inflammation caused by LPS (121) (130). Oxidative stress is another factor leading to inflammatory response. Astragaloside IV can reduce the level of oxidative stress through AMPK/SIRT1 and PI3K/AKT signaling pathways. Dendrobium, Mulberry fruit polysaccharide and Ganoderma lucidum extract have antioxidant capacity, which can reduce the level of malondialdehyde (MDA) and increase the content of superoxide dismutase (SOD), catalase (CA T) and glutathione (GSH) (116, 120, 127). The anti-inflammatory effect of traditional Chinese medicine may be mediated by increasing the abundance of anti-inflammatory bacteria Akkermania, Parabolides, Lactobacillus, Bacteroides and Blautia (141, 151).

### 5.3 SCFAs

TCM affects T2DM and NAFLD by affecting the abundance of SCFAs producing bacteria and the metabolism of SCFAs. SCFAs (acetate, propionate and butyrate) are produced by selective fermentation of intestinal microorganisms (154). Acetate participates in host energy metabolism by promoting the secretion of intestinal hormones (GLP-1 and PYY). Acetate is mainly produced by bifidobacteria and lactobacillus (155). Acetate can be converted to butyric acid by Firmicutes bacteria. Butyrate can protect the intestinal barrier and reduce inflammation (156). Clostridia, Bacteroides and Bifidobasteria are related to the production of butyric acid (157). Propionate is believed to reduce fat production, and serum cholesterol level has a beneficial effect on disorders of lipid metabolism (158). Green Tea Polyphenols increased the levels of acetic acid and butyric acid, which may be related to the increase of Clostridium populati, Blautia luti, Akkermania muciniphila and Thiothrix unzii (125). The increase of SCFAs content in Pueraria lobata star may be due to the increase of the content of Lactobacillus, Bifidobacterium and Turicibacte (135). Using Inulin in NAFLD treatment, it was found that SCFAs were positively correlated with Bacteroidetes, Akkermania and Bifidobasterium, and negatively correlated with Proteobasteria, Blautia and Ileiberium (122). In addition, the study also found that ginsenoside Rg1 can increase Lachnospiracea_ NK4A136_ The proportion of group, Roseburia and Romboutsia increases the content of SCFAs (98).

### 5.4 Bile acid metabolism

Bile acid metabolism, as an important part of the body’s regulation of glucose and lipid metabolism, is mainly mediated by G-protein coupled BA receptor (TGR5) and nuclear receptor Farni X receptor (FXR) (159). TGR5 is expressed in intestinal epithelial cells. The activation of TGR5 is conducive to the renewal of intestinal epithelial cells and the repair of intestinal barrier function (160). Cholesterol - 7 α- Hydroxylase (CYP7A1) is the rate limiting enzyme for converting cholesterol into BA (161). The changes of intestinal flora involved in bile acid metabolism mainly include bile salt hydrolase (BSH) and α- Dehydroxylated genera decreased and taurine metabolism related genera increased (137). Radix Scutellariae, Cinnamaldehyde, Astragaloside IV, Morchella esculenta mushroom polysaccharide, Nuciferine and Simiao Wan can all improve glycolipid disorder through bile acid metabolism. Detailed mechanisms are shown in **Tables 1** and **2**.

## 6 Conclusion and prospect

Traditional Chinese medicine has the potential to treat metabolic diseases such as diabetes and non-alcoholic fatty liver. Reshaping intestinal flora and regulating intestinal microbial metabolism is the key for traditional Chinese medicine to play a therapeutic role. at the same time, intestinal flora also provides a new opportunity to clarify the mechanism of traditional Chinese medicine in the treatment of diseases. The main mechanisms of traditional Chinese medicine include: improving the proportion of thick-walled bacteria and Bacteroides, increasing dominant flora and reducing harmful flora; regulating intestinal microbial metabolites such as short-chain fatty acids and bile acids; and restoring intestinal barrier. Increase the expression of tight junction proteins and reduce the level of inflammatory factors. It can be seen that maintaining the stability of intestinal microecology is of great significance to human health. The intestinal microecology is stable and healthy, and the destruction of intestinal microecology leads to the occurrence of disease. Lactobacillus acidophilus, Streptococcus thermophilus, Lactobacillus bulgaricus and/or Bifidobacterium can improve blood glucose levels in patients with diabetes. It can be inferred that dietary fiber, probiotics and probiotics are beneficial to the recovery of the disease. In addition, fecal microorganism transplantation has therapeutic potential in chronic inflammation, functional bowel disease, insulin resistance and morbid obesity. Herbs can be used as a treasure trove of potential probiotics for more in-depth research.

## Author contributions

All authors contributed to the article and approved the submitted version.
